# The “Grass-Fed” Milk Story: Understanding the Impact of Pasture Feeding on the Composition and Quality of Bovine Milk

**DOI:** 10.3390/foods8080350

**Published:** 2019-08-17

**Authors:** Mohammad Alothman, Sean A. Hogan, Deirdre Hennessy, Pat Dillon, Kieran N. Kilcawley, Michael O’Donovan, John Tobin, Mark A. Fenelon, Tom F. O’Callaghan

**Affiliations:** 1 Department of Food Chemistry & Technology, Teagasc Food Research Center, Moorepark, Fermoy, P61 C996 Cork, Ireland; 2 Teagasc, Animal & Grassland Research and Innovation Centre, Moorepark, Fermoy, P61 C996 Cork, Ireland; 3 Department of Food Quality & Sensory Science, Teagasc Food Research Center, Moorepark, Fermoy, P61 C996 Cork, Ireland

**Keywords:** dairy farming, pasture, milk, TMR, nutrition, grass-fed

## Abstract

Milk is a highly nutritious food that contains an array of macro and micro components, scientifically proven to be beneficial to human health. While the composition of milk is influenced by a variety of factors, such as genetics, health, lactation stage etc., the animal’s diet remains a key mechanism by which its nutrition and processing characteristics can be altered. Pasture feeding has been demonstrated to have a positive impact on the nutrient profile of milk, increasing the content of some beneficial nutrients such as Omega-3 polyunsaturated fatty acids, vaccenic acid, and conjugated linoleic acid (CLA), while reducing the levels of Omega-6 fatty acids and palmitic acid. These resultant alterations to the nutritional profile of “Grass-Fed” milk resonate with consumers that desire healthy, “natural”, and sustainable dairy products. This review provides a comprehensive comparison of the impact that pasture and non-pasture feeding systems have on bovine milk composition from a nutritional and functional (processability) perspective, highlighting factors that will be of interest to dairy farmers, processors, and consumers.

## 1. Introduction 

Milk is a highly nutritious food and valuable source of minerals, fats, amino acids, and vitamins, that help individuals meet their recommended daily intake of essential nutrients [1,2]. There are a variety of feeding systems used to sustain dairy or meat production animals across the globe. The system used is driven by economic considerations to increase profitability, as well as environmental and climate factors, land availability, and energy requirements of the animal. Traditionally, dairy farming consisted of outdoor grazing which relied on ruminants to convert fibrous materials into valuable food products such as meat and milk. However, the intensification of farming systems, the progressive mechanization of agriculture, and an improved understanding of animal nutrition has led to the development of indoor feeding systems using optimized total mixed ration (TMR) diets. Such systems have become prevalent in Europe and North America for large dairy herds since the 1950s [3,4]. Despite the reduction in global dependency on forages, grass-based dairy production remains the key agricultural industry in some developed countries with a suitable climate, particularly Ireland and New Zealand [5]. Pasture-feeding is practiced in Ireland due to its fertile soils, temperate climate and abundant rainfall that favor grass growth throughout most of the year and provides ruminant production systems with a cost-effective, high quality feed source [6]. 

Research has demonstrated that there is a 2.5 c/L reduction in milk production cost for every 10% increment in the proportion of grass included in the overall diet of a dairy cow [7]. These findings provide the Irish dairy farming industry (primarily grass-based) with a competitive edge over other milk producing countries [8]. With that, a recent study has demonstrated that on a fresh matter basis grazed pasture constitutes the largest component of the Irish cow diet, typically accounting for 96% of the diet, equating to approximately 82% of dry matter intake [9]. 

Milk composition is affected by several factors including animal breed, age, health status and stage of lactation [10]. Research has shown that significant variation in the milk macronutrients (fat and protein) and micronutrients (e.g., amino acids (AA), minerals, vitamins and fatty acids (FA)), caused by differences in the feeding regimen also results in alteration to functional and sensory characteristics of dairy products [11]. For example, significant increases in the concentrations of conjugated linoleic acid (CLA) and unsaturated fatty acids were reported in milk derived from pasture-fed cows compared with that from TMR-fed cows [12]. Diet is a primary factor that can be targeted to manipulate the composition and the nutritional status of bovine milk [13]. In the past, a major objective of dairy research has been to increase milk yield and production efficiency of cows, however, requirements of the industry today demand that this is done while considering impacts on animal welfare, sustainability and the nutritional profile of the final product [11,14]. 

A major aspect of a pasture-based feeding system is that it typically results in a seasonal dairy supply. With that, to maximise grassland utilization, cows are strategically calved over a short period of time at the beginning of the season (i.e., in Ireland: February–March). This is done to allow cows to move outdoors from winter housing and begin milk production coinciding with the grass growing season. While there are a number of benefits to this, in terms of utilisation of grassland as a cheap source of excellent feed, profitability and sustainability, a seasonal calving system results in some added challenges for the industry’s milk processors and manufacturers. Such challenges include a seasonal milk supply with periods of “peak” milk production, after which milk yield of the cow declines in the latter parts of the year, as cows transition from the mid to late stages of their lactation. During this transition, milk composition also changes. Olde Riekerink, et al. [15] demonstrated that seasonality can have an effect on the somatic cell count of milks and noted that *Streptococcus uberis* incidence rate of clinical mastitis seemed to be associated with being on pasture, whereas *E. coli* incidence rate of clinical mastitis was more housing related. Kristensen, et al. [16] also reported that cows who had increased time with access to pasture had reduced milk somatic cell counts compared to cows with reduced pasture access. O’Callaghan, et al. [17] demonstrated the effect of lactation in cows fed TMR or pasture-based diets on milk composition, whereby between the mid and late lactation periods, milk fat and protein concentrations increased, while milk lactose concentration decreased. Gulati, et al. [18] also demonstrated the changing concentration of the mineral composition of milk coinciding with transition from mid to late lactation periods. Such changes in milk composition can pose added challenges to processors and manufactures in terms of changing milk intake volumes, processability of milks, formulation of nutritional beverages and changes to milks functionality and characteristics. As such, to overcome these challenges, certain products are produced on a seasonal basis. 

This review provides a comprehensive overview of the implications the choice of feeding system (pasture and TMR) has on the composition, characteristics, and quality of bovine milk and dairy products. 

## 2. Nutritional Value of Milk

**Figure 1:**
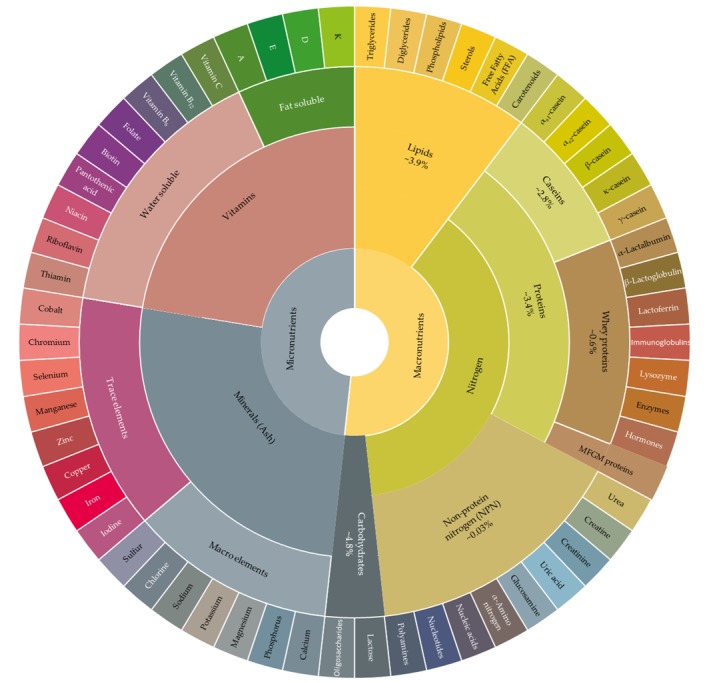
Bovine milk composition wheel. Sections are not proportionate to the actual content of each component in milk. Concentrations of these components in milk are provided in Table 1.

Milk is a nutrient-dense food that provides mammalian neonates with the required nutrients for growth and development. Being the primary source of nutrition for developing infants, the complex mixture of fat and water-soluble components, i.e., proteins (caseins and whey proteins), carbohydrates (mainly lactose), minerals, and vitamins (Figure 1 and Table 1), varies to address the nutritional and energy needs of different species [19]. Moreover, the milk content of these constituents can vary within the same species due to individual differences between animals and breed, with animal diet and stage of lactation playing an essential role in this variation [20].

Although bovine milk and dairy products are an excellent source of protein, vitamins, and calcium (Ca), a growing number of individuals have adopted a dairy-free diet due to suggested associations between milk and dairy products with coronary diseases (e.g., cardiovascular diseases, hyperlipidemia, etc.) and weight gain (obesity) [1]. Recent studies have shown this association not to be true [21,22]. In addition, meta-analysis studies [23,24] have reported the beneficial effect and inverse association with dairy consumption and CVD [25]. Similarly, the association between the consumption of butter and CVD has been a matter of debate recently with an increasing number of systematic reviews and meta-analyses suggesting a weak or neutral association of butter consumption and CVD [26,27,28], therefore future studies to conclusively elucidate the relationship of butter consumption and risk of CVD are required. 

Daily consumption of 0.5 L of milk or an equivalent amount of other dairy products supplies a significant amount of various essential nutrients that are required on a daily basis [2]. The decision to follow a dairy-free diet may be driven by potential intolerance to one or more of milks’ components such as lactose [29], which as an example is often driven by assumption rather than confirmation through testing [26]. However, when consumers abstain from dairy products entirely without adequate substitution, their risk of developing nutritional complications and deficiencies such as Ca, vitamin D or long chain *n*-3 (Omega 3) FA may increase [29,30,31]. Statistics have demonstrated that two thirds of the world population suffer from lactose intolerance with variations present both between and within countries [32]. Nevertheless, innovations in dairy processing have resulted in milks with modified compositions such as low or fat free milk, and lactose free milk developed to target specific demographics and accommodate the dietary requirements of various consumers [30]. Furthermore, with the increased demand for “natural” foods, several studies have highlighted the feasibility of altering milk composition at the farm stage through dietary intervention to control the protein and fat content as well as the FA profile, without the need for mechanical modification (i.e., processing, fat separation, ultrafiltration etc.) [20,33,34]. 

## 3. Milk Fat

Milk contains an array of substances that are biologically active and provide immunological protection to neonates [45]. Fat is one of the most valuable components of milk. From a functional point of view, fat plays a role in consumer preference of dairy products, especially when it is a major constituent of the dairy product, e.g., butter, cream, and full fat cheese. Furthermore, fat can be a source of either desirable or undesirable flavor traits in milk and dairy products, primarily because a large portion of volatile flavor compounds are fat-soluble. The composition of milk fat can significantly affect the physiochemical properties of dairy products (e.g., hardness, spreadability, melting, processability, etc.), and the percentage fat content positively correlates with the highly satisfying smooth and silky mouthfeel, and thus to the overall quality of the final product. 

Milk fat is comprised of approximately 400 different FA of various chain lengths, most of which are saturated fatty acids (SFA), with a lesser amount of mono unsaturated fatty acids (MUFA) and poly unsaturated fatty acids (PUFA) (2%–5%) making its composition very complex [46,47]. Trans FA and SFA have been a concern to consumers. These negative perceptions have been found inaccurate with the increasing scientific evidence that a number of milk FA such as *trans* vaccenic acid and linoleic acid isomers have demonstrated a positive effect on health and against diabetes, obesity and metabolic syndrome [28,48]. Consequently, there has been a growing interest in increasing these nutrients content in milk [49,50]. In addition to CLA, milk fat contains important types of Omega-3 (*n*-3) FA such as eicosapentaenoic (EPA) and docosahexaenoic (DHA) acids that are essential for normal physiological functioning and human health [51]. Studies have also demonstrated cholesterolemia-lowering, antibacterial and anticancer properties among numerous health beneficial factors of other milk fat components including the bovine milk fat globule membrane (MFGM) and its phospholipids [52].

## 4. Impact of Feeding System on the Lipid Fraction of Bovine Milk

Milk lipid fraction is primarily comprised of triglycerides (~98%), with diglycerides, phospholipids, free fatty acids and other lipophilic molecules (e.g., β-carotene, vitamins and terpenes) comprising the remaining proportion (Figure 1). Fat material present in globular form–being in an aqueous medium–are shielded by a complex lipoproteinaceous structure known as the Milk Fat Globule Membrane (MFGM), which protects the fat globule contents from oxidation and lipolysis after secretion from the mammary gland [36]. Inside the globule, FAs are present in various chain lengths and degree of saturation. Approximately 50–70% of these FAs are long chain FA (C ≥ 16); 30–50% are short (4 ≤ C ≤ 8) and medium length chain FAs (10 ≤ C ≤ 14). These FAs are esterified on the di and triglycerides of the milk lipid fraction or may exist as free molecules (mostly volatile due to their low molecular weight) in the headspace [34,37]. Note that this esterification process does not occur randomly, rather, it is specific to the type and chain length of FA [36]. For instance, while butyric (C4:0) and caproic (C6:0) acids (short chain FA) are esterified at *sn*-3, medium length chain FAs are preferentially esterified at *sn*-1 and *sn*-2 positions of the triacylglycerol molecule. On the other hand, stearic acid (C18:0) (long chain FA) is selectively placed at *sn*-1 position, whereas oleic acid (C18:1) preference is *sn*-1 and *sn*-3 positions [47]. This esterification process is also species dependent. For instance, while palmitic acid (C16:0) is predominantly esterified at *sn*-2 (60%–70%), in bovine milk C16:0 is esterified at the outer positions of the triglycerol molecules in human milk [53,54]. 

Two sources of milk FA in bovine milk have been identified: feed (animal’s diet) or as products of digestive and metabolic processes in the rumen [47]. Long chain FA are typically derived from dietary sources and they reach the milk via the bloodstream, while the short and medium chain FA (C4 to C14 and some C16) are produced in the mammary gland by de novo synthesis from precursors such as acetate and butyrate [34,55,56,57]. Acetate produced from carbohydrate fermentation in the rumen in addition to the β-hydroxybutyrate produced from absorbed butyrate by the rumen epithelium represent the carbon source for the synthesis of these FA in the ruminant’s mammary gland (i.e., de novo synthesis) [55]. The levels and types of de novo synthesized FA vary, and the process is controlled by a number of key genes that are expressed in the mammary gland during milk production [58]. Due to the relatively rapid response of the lipid fraction to changes in diet in comparison to other milk constituents, feeding system will have a pronounced effect on the milk fat content and composition (i.e., FA profile). These changes occur either through introducing FA that are directly derived from the feed material, through alteration of the rumen conditions (e.g., pH), or via dietary source by providing substrate FA that are biohydrogenated in the rumen to other FA (e.g., C18:3 *n*-3 breaks down to α-linolenic acid, vaccenic acid and CLA and other FAs) [47,59]. 

The potential health benefits of FA in the milk lipid fraction have been reviewed [2,60]. The authors reported that molecules such as PUFA, MUFA and other FA that are present in milk showed biological significance by exerting antimicrobial, anti-carcinogenic and anti-inflammatory activities, as well as regulatory effects on blood serum lipid profile. Therefore, increasing their concentration in milk would enrich milks’ nutritional profile. High proportions of α-linolenic acid (C18:3 *n*-3) is present in fresh pasture and a number of oilseeds such as linseed; while linoleic acid (C18:2) predominates in corn silage, cereals and other oilseeds [61,62,63]. As these FA represent the most abundant *n*-3 and *n*-6 fatty acids in milk, the ratio between linoleic acid (*n*-6) and α-linolenic acid (*n*-3) concentrations is considered an indicator for nutritional impact of milk fat on human health (i.e., lower *n*-6:*n*-3 ratio is beneficial for human health) [64]. These dietary PUFA cannot be synthesized by humans or animals and so must be sourced from the diet. In addition to α-linolenic acids’ role as precursors of CLA, it increases the n-3 FA to achieve an improved *n*-6:*n*-3 ratio in human nutrition [63]. 

Studies comparing the effect of pasture versus TMR feeding systems on the milk fat content have demonstrated that milk produced from TMR systems has a lower or depressed milk fat content [65,66,67,68,69,70]. This observation has been attributed to the high fiber content of a pasture diet in addition to the abundance of materials that induce fat synthesis in the mammary gland, as opposed to TMR diets that differ from fresh pasture systems in their starch content. As a consequence of higher starch content in TMR, the feed fiber content decreases causing the rumen bacteria to produce large amounts of propionate. Moreover, the availability of excessive fermentable energy can cause a drop in the rumen pH, resultant from an increase in the molar concentration of volatile fatty acids (VFA) which favors the synthesis of trans FA [34]. Higher starch content (i.e., energy) in TMR diets also affects the biohydrogenation rate of the unsaturated FA in the cow rumen reducing their concentrations in the milk. Diets with high starch and unsaturated fat content have been associated with reduced milk fat concentrations [57]. This reduction in milk fat concentration occurs due to the suppression of *de novo* synthesis of FA which limits the milk fat content of short and medium chain FA but not long chain FA as the latter are readily derived from the diet [34,56]. Such diets (high in starch and low in fiber) also increase the risk of subacute ruminal acidosis (SARA), a well-recognized and economically important digestive disorder that is incurred by over feeding grain diets where daily episodes of low ruminal pH occur when ruminal pH stays between pH 5.2 and 6 for a prolonged period [71]. In addition to the considerable economical loss associated with SARA, this can also cause impaired animal welfare and predisposition to other diseases [72]. 

It has been found that when given the opportunity, dairy cows exhibit a natural tendency towards spending considerable time outdoors during night time under the relatively mild weather conditions, with a greater preference for a large pasture than for a small outdoor sand pack [73]. In Ireland, cows usually graze outdoors from the middle of February till late November. With that (i.e., pasture feeding) allows the cow to express normal behaviors such as grazing [74,75]. Furthermore, outdoor feeding systems can be beneficial for udder, foot and leg health [76,77,78]. From an environmental perspective, pasture feeding systems are efficient in their use of resources through grassland and soil management [74]. O’Neill, et al. [79] have found that pasture feeding systems have less impact on the environment by lower production of enteric methane (CH_4_) emissions/cow relative to DMI and unit of milk fat and protein yield compared to that of a TMR system. The greater environmental impact of TMR (confinement) system is related to the increased use of concentrate feed and the longer manure storage period [80].

Significantly higher percentages of health promoting FA have been recorded in milk fat derived from pasture feeding [81]. Using data from 29 published experiments on Holstein cows and 120 dietary treatments, Moate, et al. [82] found a substantial variation in the concentration of individual FAs in milk derived from pasture vs TMR feeding systems. The study found that total FAs, total *de novo* FAs, total C16:0 and total preformed FAs were numerically but not statistically higher in milk derived from cows fed pasture compared with those fed TMR diets. Moreover, in the same study, concentration of C15:0, C17:0 and the estimated mean concentration of total *trans* C18:1 FAs were significantly higher in milk derived from pasture fed cows compared with TMR fed cows. Moate, et al. [82] have also suggested that the variation in the concentrations of individual milk FAs is influenced by various dietary or animal factors.

While TMR feeding has resulted in increased concentrations of SFA, milk fat from pasture feeding has a lower content of SFA and a higher content of unsaturated FA with a higher contribution of PUFA [70]. Pasture feeding systems, or the inclusion of fresh pasture in the cows’ diet can lower the *n*-6:*n*-3 ratio of milk fat [83] and increase the milk fat content of an array of beneficial MUFA and PUFA (*n*-3) and their isomers. Such nutrients include but are not limited to vaccenic acids (*trans*-11 C18:1), α-linolenic acid (C18:3), *cis*-9,*trans*-11 CLA, *trans*-11,*cis*-9-18:2 providing a nutrient-rich milk with an improved thrombogenicity index compared to milk from a TMR system. Milks from a TMR system on the other hand have higher content of SFA of various chain length (C12:0 to C16:0) that are known for their potential contribution to hypercholesterolemia and subsequent health problems [12,84,85,86,87,88] and Omega 6 fatty acids. This pattern for alteration of milk fatty acids has been confirmed in a variety of dairy products produced from pasture milk such as cheese (e.g., Asiago, Yak, Cheddar, Feta) [89,90,91,92,93] and butter [81,94,95] when pasture and TMR feeding systems were compared. This can be attributed to the high supply of these nutrients and FA substrates through fresh pasture, and a high delivery of these FA to the mammary gland which enhances their final concentrations in milk [96,97]. 

A reverse trend between C18:0 and C18:1 (*cis* and *trans*) and C16:0 (positively correlated with milk fat hardness) was observed in French milk where the former FA increased in spring and summer, while C16:0 increased in winter [98]. These observations can be attributed to the herd management systems applied in that region, where pasture feeding is applied during spring and summer and TMR feeding is applied during the winter period. Moreover, and apart from seasonal changes, an increase in CLA concentration in milk fat has been observed due to the supplementation of grass silage with rapeseed oil. This was associated with an increase in the concentration of MUFA and PUFA and a decrease in the concentration of short chain, medium chain and SFA [99] confirming the reverse relationship between the two classes of FAs referred to previously. 

This contrast in the composition of milk lipid fraction, particularly the FA profile of the pasture vs TMR milks extends beyond variation in concentrations and percentages to affect the milk fat processability and the characteristics (physiochemical and sensory) of the final product. When milk derived from these feeding systems are manufactured into fat containing dairy products, a significant effect on consumer preference is observed. It has been highlighted that the concentration of unsaturated FAs and CLA in milk fat correlates negatively with that of SFA, and this relationship affects the texture (softness/hardness) of dairy products such as butter [94,95,99,100]. When studying the effect of pasture vs TMR systems on the FA and sensory profiles of Caciocavallo cheese, Esposito, et al. [101] found that the sensory panelists were able to distinguish both cheeses produced from pasture or TMR feeding systems. Cheeses produced from ‘pasture milk’ were reported to have higher ratings for a number of favorable sensory attributes such as texture (friability and graininess), which the authors attributed directly to the variation in the FA profiles in the milks. Butter and cheese produced from ‘pasture milk’ were yellower in color as a result of the higher β-carotene concentrations in milk, had reduced hardness and rancidity scores at room temperature, and higher preference for various attributes such as creaminess, appearance, flavor and color [81,89,93,94,102,103]. A linear increase in the proportion of unsaturated FA and a decrease in the proportion of SFA are associated with increasing the portion of fresh pasture in the cow diet which impacts the textural properties of milk fat. The ratio of C16:0 (palmitic acid) to *cis*-9 C18:1 (oleic acid), is referred to as the spreadability index, whereby increased oleic acid results in the production of a softer, more spreadable butter [94,96,99]. Meanwhile, pasture feeding is expected to improve the organoleptic and some physiochemical traits of butter as reported by Radkowska and Herbut [104]. Butter produced from milk derived from pasture feeding is characterized by higher iodine number, acid number and peroxide and thiobarbituric acid (TBA) values. This is resultant from the degree of unsaturation in the FA composition of butter. Therefore, despite the positive effect of pasture feeding on processability, aroma, color and taste, increased unsaturated FA content can increase the susceptibility of butterfat to undesirable quality changes such as lipolysis and oxidation [104]. However, the high levels of natural antioxidants such as tocopherols and carotenoids transferred into the milk fat from fresh pasture can also be associated with increased oxidative stability (low peroxide and TBARS values), as shown in a study that evaluated the physiochemical traits and sensory quality of 8 commercial Azorean butter brands [105].

## 5. Potential Methods for the Verification of Pasture or “Grass-Fed” Dairy Products

Fatty acid profiling of milk could serve as an indicator of the feeding system used during milk production. Ruminants synthesize very little C18 FA which are almost exclusively of dietary source, therefore, it is hypothesized that the high percentage of unsaturated FAs (e.g., linolenic acid as a predominant *n*-3 FA) in fresh pasture milk could potentially be used as a biomarker for the verification of pasture feeding system [106,107]. However, identifying milk from cows fed fresh grass indoors (stabled cows) vs that from outdoor grazing has proven difficult [106]. In addition, other than serving as a health indicator, the *n*-6:*n*-3 ratio discussed earlier was also used as a quality indicator to distinguish pasture-fed lamb [108] which raises its potential to differentiate between milk derived from pasture and TMR feeding systems. Advances in research coupled with multivariate data analyses have been providing promising tools to distinguish pasture-fed from TMR products including FA profiling and quantitative nuclear magnetic resonance (^1^H-NMR) [59,109]. Sundekilde, et al. [110] reviewed recent advances in ^1^H-NMR based milk metabolomics applications and discussed the applications of NMR spectroscopy in investigating properties of milk fat and protein fraction. Furthermore, a number of advantages of ^1^H-NMR were highlighted including it being a non-destructive method of analysis with minimal sample preparation, and the ability to detect all mobile hydrogen-containing molecules increasing its capacity to be used for verification of milk purposes. 

## 6. Milk Protein

Milk is an excellent source of high-quality protein (~3.5 g/100 mL) as it contains the 9 essential amino acids humans require within its structure [111]. Protein occurs in milk in two major groups: Caseins (α_s1_, α_s2_, β, κ, and γ caseins), and whey proteins (α-lactalbumin, β-lactoglobulin, lactoferrin, immunoglobins, lysozyme, enzymes, and hormones) (Figure 1 and Table 1). Similar to any other dietary protein, milk proteins serve as a primary source for indispensable AA and organic nitrogen that is important for growth and development [45,112]. Milk protein bioactive peptides have been studied intensively and a number of these peptides were shown to possess antihypertensive, antithrombotic, immunomodulating and antimicrobial properties along with many other biological properties that are beneficial for human health [113]. Due to their positive impact on body functions, specific milk protein fragments are considered important bioactive peptides in reducing the risk of obesity and the development of type two diabetes [114,115]. More interestingly is the potential of several milk constituents including proteins to influence immune and neural networks affecting infection rates and mood [116]. These bioactive peptides (fragments) are generated during fermentation by dairy starter cultures, and they have been found in a number of dairy products such as cheese (during ripening) and fermented milks [117,118]. Future work to examine if cow feeding system has an effect on the content or presence of bioactive peptides in milk would be beneficial in this regard. On the other hand, the ingestion of milk proteins for some individuals might result in the occurrence of an altered or abnormal reaction called cow milk allergy. Cow milk proteins can cause hypersensitivity to susceptible individuals and is related to the genetic polymorphisms of milk proteins which can elicit allergic reactions of different degrees [119]. 

In addition, components of protein are also present in the milk fat globule membrane (MFGM) as lipoproteins. Caseins comprise the vast majority (~80%) of the proteinaceous material in bovine milk, while whey proteins make up the remaining part (~20%) [111]. These ratios can differ between species (i.e., human, goat, sheep, etc.) [111]. Milk protein is a decisive factor in the milk payment scheme, because it is the foundation of many products particularly cheese and yoghurt. Therefore dairy farmers take measures to ensure that the lactating cows diet meets the required nutrient dry matter intake in order for them to maintain proper body condition and produce milk with adequate protein yield, particularly in countries where the dairy producers are paid more than double the price for protein as that for fat [120]. In essence, the ultimate goal is to increase protein content (%) while also maintaining or increasing the protein yield (kg/day) [121].

## 7. Impact of Feeding System on Bovine Milk Proteins

Protein synthesis is one of so many complex physiological processes that occur in the mammary gland and requires vast amounts of energy. Protein production can vary considerably between and within the various genetic groups of cows [122]. Although these genetic differences between breeds exist, feeding system has also been shown to impact milk and protein yield [123]. A link between protein synthesis and the feeding system was established stemming from the fact that the animal diet readily alters the AA content and function of the rumen [55]. The rumen thus plays an essential role in that its metabolic products can influence the process of protein synthesis in the mammary gland and consequently milk protein yield O’Callaghan, et al. [59] observed several qualitative and quantitative differences in the ruminal content of lactating dairy cows fed diets of perennial ryegrass or a perennial ryegrass with white clover sward compared to those fed TMR diets. O’Callaghan, et al. [59] showed that both L-lysine and methionine presented at higher average concentrations (μM) in the rumen of cows fed diets of perennial ryegrass and white clover or perennial ryegrass only compared to TMR diets. This could be due to the high content of methionine in pasture compared to silage and hay as reported by Villeneuve, et al. [96]. This variation in AA concentration translated into higher protein yield and casein content with an improved protein quality in milk derived from pasture vs TMR feeding systems [17,18]. The enhanced protein yield and casein content are ascribed to the crucial role of specific AA (e.g., methionine and lysine) in the process of protein synthesis [124,125,126,127].

Couvreur, et al. [94] found that milk protein yield and milk protein content increased linearly as the ratio of fresh grass to corn silage increased. The authors attributed this to what they referred to as the “specific effect of grass” rather than to energy deficit, or the interaction induced by the proportion of fresh grass in the diet, noting that all the diets in the study were formulated to provide the same energy, protein and dry matter intake. The study interpreted the “specific effect of grass” as the linear increase in the propionic acid content in the rumen, which increased milk and protein synthesis, thus the higher protein yield and content. 

Diets of high quality improve the rumen function and the efficiency of milk production, yielding higher milk and protein as well as higher fat and milk protein contents [128]. On the other hand, feeding low quality pasture would affect dry matter intake resulting in decreased milk, protein, and casein yields as reported by Mendoza, et al. [87]. Given that pasture quality as well as pasture protein degradability can be inconsistent and vary over time depending on season [64,129,130] compared to TMR, this should be a point to consider when studying the impact of pasture and TMR feeding systems on milk protein. Furthermore, differences in the pasture composition related to the diversity in the botanical species and plant growth stage can cause variations in the chemical and nutritional characteristics of pasture [101] and significantly impact the yield and composition of milk [131]. In an attempt to investigate the differences in the chemical composition of milk from grass-fed cows under two different farm systems (organic or conventional), Schwendel, et al. [132] reported higher amounts of total α_s1_ and κ caseins in organic milk vs higher amounts of β-casein and β-lactoglobulin in conventional milk. The authors attributed these compositional differences in milk protein to differences in pasture composition (white clover was 50% higher in the organic milk farm). Clover content in the cows’ diet has been associated with increased casein content, protein and milk yield due to an increase in the feed intake compared to ryegrass [131,133,134]. It is noteworthy that there is often a misconception about “organic” as this may not always mean pasture or grass fed in the context of dairy, whereby organic TMR diets can be formulated. While the term organic refers to the means by which products are produced, differences in composition can be attributed to the type of feeding system (i.e., pasture feeding vs TMR) as opposed to just the “organic” production alone. Barłowska, et al. [135] also found that although milk derived from TMR feeding system had a higher yield with higher fat and protein, milk acquired through pasture feeding had an improved processability, protein:fat ratio and higher content of whey proteins (particularly β-lactoglobulin and lactoferrin) which improves the bioactive status of pasture milk given that lactoferrin and its peptides such as lactoferricin and lactoferramin are biologically active compounds in milk of high importance due to their positive impact on human health [136,137,138]. 

These qualitative and quantitative variations in the subcomponents of milk protein due to dietary variations could be used to explain differences in the physicochemical properties of milk including the higher ethanol stability, increased heat stability and shorter rennet-induced clotting time of milks derived from pasture vs TMR feeding systems [104,135]. Although no significant difference was reported in rennet cheese yield per 10 L of milk, Radkowska and Herbut [104] found that cheese curd (rennet-coagulation) produced from milk derived from pasture feeding was firmer, contained significantly higher solids content and lower water content compared to that produced from milk that was derived from TMR feeding. Furthermore, the same group reported significantly higher protein and carbohydrate contents in pasture cheese and observed that TMR cheese was harder to process as a result of its markedly loose consistency compared to the pasture curd. The latter which received higher scores for its consistency when evaluated by expert sensory panelists. 

Although quality pasture contains considerable amounts of metabolizable protein and AA [139,140] which could reach double the recommended levels for early lactation in some countries [141], its low content of rapidly fermentable carbohydrates (i.e., non-fiber carbohydrates such as starch and pectin) limits the supply of dietary energy and consequently constrains high milk and protein yields in pasture-only based diets [141,142,143]. This could contribute to the higher protein yield and/or content from milk derived through TMR compared to that of pasture based systems in some studies [69,144,145,146,147]. Rapidly fermentable carbohydrates increase the production of glucose, propionate and microbial protein which provides substrate for the cow to produce more milk and milk protein [121] due to the higher energy supply per unit [148]. Furthermore, the lack of sugar-rich feed in pasture-based diets that stimulate the production of butyric acid used for protein synthesis, could be another reason for the lower protein content in milk from such feeding systems [149].

Based on a series of experiments using a wide range of feedstuffs, Stockdale [150] suggested the use of starch-based supplements such as cereal grains and compounded concentrates to increase the metabolizable energy intake of pasture grazing cows as an excellent way to increase milk protein content by 1 g/kg. As such, this leads to the production of large amounts of volatile FAs and propionate in the rumen. The authors also suggested increasing the quantity of consumed pasture or using maize silage as a supplement to increase the metabolizable energy intake and invoke similar response in milk protein content. Note that the availability of excessive dietary energy is not necessarily the answer to increasing milk protein, on the contrary it may have a negative effect on the milk protein yield as reported by Cannas, et al. [151]. 

Feed intake and the amount and source of energy and protein in the diet (availability of N) are important factors to consider as they affect the ruminal fermentation, determine the amount of microbial protein synthesized in the rumen and the flow of microbial and dietary protein to the small intestines [148]. Pasture contains high levels of highly degradable crude protein (20–30%) [141,152,153,154] and low levels of nonstructural carbohydrates which increases the intake of crude protein and lowers the efficiency of N utilization by the grazing cow [129,142,155]. Overfeeding highly degradable crude protein to the extent that exceeds microbial needs will result in the production of large amounts of NH_3_ that will be converted to urea increasing its concentration in the rumen, blood and milk. As such this reduces the efficiency of N conversion to microbial protein in the rumen and consequently compromises the supply of protein to the small intestine [130,142,143,156,157,158,159]. Conversion of NH_3_ to urea is an energy utilizing process that puts extra metabolic strain on a system that is already limited by energy supply [142]. This explains the strong correlation between milk urea concentrations with protein:soluble carbohydrates ratio in pasture, which is also found to correlate negatively with milk fat and milk protein production [141,153,154]. Unlike TMR diets where the efficiency of dietary N to milk protein conversion is high, the high ratio of highly degradable N in pasture to its fermentable energy reduces the efficiency of N conversion to microbial protein [68]. Due to this reduced efficiency of N conversion to microbial protein, some AAs would be used to compensate for the lack of energy (i.e., used as a glucose precursor) rather than milk protein synthesis [160], which although may not affect protein content significantly, may reduce protein yield [68].

## 8. Impact of Feeding System on the Micronutrients and Lactose content of Bovine Milk

In addition to the macronutrient component of milk, micronutrients such as vitamins and minerals are also present in milk and are of essential importance for the development of the neonate particularly during the first month of life [161]. Other bioactive compounds of a lipophilic nature are affected by the cow feeding system in a manner that is similar to milk lipids. Fresh pasture as a plant material (e.g., perennial ryegrass, white and red clover) is a good source of various vitamins and antioxidants that are transferred through complex digestive processes to the mammary gland and then to milk, which explains the higher content of compounds such as β-carotene, terpenes, lutein, Vitamins A (retinol), E (tocopherol), and phytol (a derivative of chlorophyll) in ‘pasture milk’ compared to ‘TMR milk’ [81,89,103,139,162,163,164,165]. The yellow coloration of milk, milk fat, and subsequently high fat dairy products is related to the β-carotene content [63,166]. Such differences in product color can affect consumer acceptance as some consumer groups associate a creamy milk and yellow butter color with natural feeding of cows [104,167]. The concentration of these bioactive compounds and their recovery rate in milk are directly related to their original dietary concentrations, thus diets rich in concentrate or corn silage would lead to a lower content of carotenoids and vitamin E compared to pasture [63]. Milk production system has also been demonstrated to have a significant effect on the fat soluble compounds of cheese [103]. Additionally, although the concentration of vitamin B_12_ in milk was positively and negatively correlated with the dietary concentration of fiber and energy-related compounds respectively. Dietary modification has a limited effect on the concentration of this vitamin; rather it is related to specific attributes of the cow [168]. This observation was contrasted by Poulsen, et al. [169] who ascribed the levels of vitamin B_12_ to the direct effect of the farm system (mainly the inclusion of grass in the diet), seasonal variation, as well as breed differences.

It is clear that the dietary concentration of the fat soluble bioactive compounds dictates their subsequent concentration in milk; therefore, a significant effect of the seasonal (botanical) variation in the pasture composition is expected. For example, Revello Chion, et al. [93] reported significant differences in the terpenoid milk profile; bioactive compounds that influence the aroma of cheese [170] and possess anti-bacterial and anti-cancer properties [171] between summer (richer in terpenoids) and winter milks that is ascribed to differences in the cow diets (fresh pasture or hays). It has been suggested that these differences can be attributed to the variation in the percentage of pasture inclusion in the diet [162]. These differences could be used to differentiate milk and dairy products of protected designation of origin (PDO) or milks derived from pastures from different terrains (e.g., valley vs mountain pastures) [170]. 

Minerals and trace elements exist in milk in an equilibrium between the soluble and colloidal phases of milk, and their content coupled with their distribution (mainly Ca, Mg, and phosphate) have essential technological significance especially in cheese making due to their direct effect on the casein stability in milk [172]. Like other milk components, their concentrations in milk are influenced by several factors such as stage of lactation, breed, animal health and environmental factors, not to mention that the overall concentration of minerals in milk is impacted by the mineral composition of the feed and soil conditions in which the pasture or corn silage (TMR diets) etc. are grown. However, the wide variation in their reported values could be attributed to analytical variation or cross-contamination during collection or processing [173], which serves as a base for understanding the contrary reports on the effect of the cow feeding system on the composition of macro and trace elements in milk. 

Milk is rich in minerals that are important for human health such as Ca, which is well known for its positive impact on bone and teeth health in addition to other biological functions including, but not limited to, nerve conduction, muscle and vascular contraction [30,173]. Minerals and trace elements exist in milk through various sources and at different concentrations. For instance, both Ca and P exist in milk attached to casein, and their contents, in addition to Mg, vary among different cow breeds and correlate positively with that of protein [169,174]. Other minerals can reach milk through supplementation in the concentrate feed or by the consumption of soil during grazing, causing significant variation in their content between conventional and organic milk [175].

In addition to the aforementioned factors influencing milk mineral content, diet and farming system could also be included; however, the impact is not clear especially with the presence of studies referring to a greater impact of the geographical conditions (i.e., soil origin and seasonal variations) and management, i.e., the inclusion of mineral supplements in the diet [176]. Gabryszuk, et al. [177] found that while Ca, Mg, and P concentrations in milk were highest in intensive production systems with no grazing (TMR) as opposed to pasture feeding farms, it is likely that grazing cows may ingest higher levels of dietary Al, increasing its content in milk. The group added that milk content of mineral elements derived through pasture systems is highly affected by the stage of plant growth and the efficiency of the soil in supplying these minerals into the plants which increase the level of variability within a short time. Gulati, et al. [18] reported that in addition to the effect of lactation period, a significant effect of animal diet on the milk mineral composition was found not only between the different feeding systems but also within different pasture-based systems. Milk that was derived from a perennial ryegrass diet had the highest mean concentrations of Ca and P, while the TMR milk had the highest concentrations of Cu and Se. Milk derived from a perennial ryegrass with white clover sward had Zn and Cu concentrations that were similar to the perennial ryegrass pasture diet and Ca and P concentrations that are similar to TMR milk. 

The variation in the supplied energy through pasture and TMR feeding systems has been highlighted in earlier sections of this review. The increased energy supplied through TMR compared to a pasture feeding system was shown to alter milk lactose content (positive correlation) especially in high starch diets when corn and fava beans are included [144,178]. Milk derived from cows fed TMR diets had significantly higher lactose concentrations compared to that derived from cows fed pasture [12,18,147]. Longer access to fresh forage within a mixed (i.e., partial TMR with different access periods to pasture) feeding system resulted in lower milk lactose concentration which is consistent with the previous studies [87,179]. Despite the consistent results of these studies, they did not provide an explanation for the effect of TMR feeding system on improving milk lactose content compared to pasture feeding. On the other hand, there are various studies which refer to either no or minimum variation in milk lactose concentration due to the feeding system, wherein fat was the main variable component and any reported variation in lactose values was due to factors other than the diet itself, such as the season or stage of lactation stage [20,169,180,181,182].

## 9. Impact of Feeding System on the Sensory Characteristics and Volatile Organic Compounds of Bovine Milk

The importance of sensory analysis comes from the fact that other analytical techniques fail to fully replicate the human response towards different stimuli (i.e., flavor, odor, etc.) [183]. As previously mentioned, animal feeding system (pasture or TMR) is shown to alter the sensory characteristics of milk and dairy products. This occurrence is demonstrated through variation in the color (yellowness of butter and milk due to milk β-carotene content) or texture (spreadability of butter, creaminess or friability and graininess of cheese) of milk and dairy products. Furthermore, the contribution of the feeding system to the FA composition (lower degree of unsaturation in milk from pasture-based diets) as well as the presence of pro-oxidants and natural antioxidants in milk are crucial for the development of oxidative changes in milk before and after processing [184]. While these differences can be differentiated through instrumentation or trained sensory panels, they could be very subtle for regular consumers to differentiate [88,185].

Compared to milk derived from TMR-diets, milk from cows fed a pasture-based diet is distinguished by the perceived grassy, cowy, mothball and barny flavor which corresponds with the presence of higher concentrations of *p*-cresol in the headspace of pasture milk. *p*-cresol is a derivative of β-carotene that is identified among the aroma compounds with the highest nasal impact frequency value in pasture milk [88,140,163]. On the other hand, milk from cows fed a TMR diet is distinguished by sweet feed/malty and sweet aromatic flavors and sweet taste characteristics [88]. Changes in the milk FA composition associated with diets can also have an effect on the sensory characteristics of milk and dairy products. For instance, alteration of cow diet can result in increasing the ratio of palmitic (high melting point temperature) to oleic acid (low melting point temperature) which might affect the perceived texture or viscosity of milk and dairy products [96,185,186]. In a study determining the effect of three diets (grass, grass/clover, or TMR) on the volatile organic compounds (VOC) and sensory properties of bovine milk, Irish assessors showed the least preference for bovine milk derived from TMR diet (overall acceptability, color, and flavor) compared to the other two. The grass/clover milk scored the highest for viscosity when compared to the two other milks [163]. Using the same diets in the previous study, O’Callaghan, et al. [81] also found that butter derived from a grass only diet scored the highest in terms of liking of appearance, flavor, and color over that from TMR, with clear statistical separation between the two grass-based diets. Butter is a high-fat product; therefore, the main contribution to differences in textural, thermal and sensory characteristics would be the variation in the FA profile that was discussed at earlier sections of the present review. 

It is well stipulated that VOCs are linked to the sensory characteristics of products particularly odors and flavors [187]. VOCs can originate in milk through the metabolic processes of the cow (e.g., rumen gases, blood, etc.) or could be infused from the animal feed into the milk which impacts the overall perceived flavor of dairy products [188,189,190]. Given that the feeding system (diet) impacts the metabolic processes of the cow, it is expected to have similar impact on the VOCs of milk and dairy products (i.e., odors and flavors) which enables the discrimination of milks from both regimens through instrumental methods coupled with statistical analyses [139,191]. Faulkner, et al. [163] found significant differences in the VOCs composition of bovine milk derived from three different diets: TMR (grass silage, maize silage, and concentrates), perennial ryegrass, or perennial ryegrass/white clover pasture diets. These changes were associated with the presence of more volatiles derived from AA metabolism (particularly branched aromatic and sulfur AA) in milk derived from pasture, due to the higher ratio of protein to readily digestible carbohydrates in pasture diets. Such changes yield a wide range of odor active compounds that can be potentially transferred to the mammary gland. Significant statistical differences in the VOCs composition of butters derived from the same feeding regimens used in the previous study were revealed [81]. These differences were found in 5 VOCs identified as acetone, 2-butanone, 1-pentenol, toluene (higher in pasture-based diets), and β-pinene. 

Studying the aroma compounds of milk from cows fed different diets, Bendall [140] found that γ-12:2 lactone (significantly odor active VOC) was detected in milk from cows fed TMR diets and absent in milk from cows fed a pasture diet. The authors of this study attributed the variation in milk flavor resulting from different feeding systems to quantitative (i.e., concentrations) rather than qualitative differences in the same set of VOCs that present in the milk regardless of the feeding system used which is in agreement with the findings of Croissant, et al. [88]. The type of forage can also lead to changes in the VOCs of milk and affect its sensory characteristics. Feeding different types of timothy grass (*Phleum pratense* L. cultivar AC Alliance) caused a significant variation in the VOCs composition in milks from cows fed three types of timothy grass namely pasture, hay or silage [96]. Milk from timothy grass pasture had higher levels of dimethyl sulfone and toluene and received high scores for grassy flavor compared to milk from cows fed timothy hay that had higher levels of γ-lactones and corresponded with higher scores for the sweet, vanilla and caramel descriptors. For timothy silage, levels of acetone, 2-butanone, and α-pinene were the highest compared to the other two milks in agreement with the results reported by O’Callaghan, et al. [81]. Furthermore, VOCs such as butanoic and 3-methyl butanoic acid; (E,E)-2,4-nonadienal; (E)-2-hexenol and 2-pentanone were associated with fatty, metallic, green, and creamy notes respectively in butter enriched with a combination of unsaturated FA and CLA [95]. Edam cheese and butter manufactured from milk derived from cows fed a cereal-based concentrate diet enriched with rapeseed oil had a softer texture (spreadable butter) with acceptable organoleptic and storage properties due to the increase in CLA concentration in this milk [99]. 

Differences in texture and appearance between Cantal cheeses obtained from milk of cows grazed on grasslands with different degrees of diversity against a control group that is fed a hay-based diet indoors underlines the importance of grazing management on milk and cheese characteristics [102]. Unlike cheese derived from hay-based diets, Cantal cheeses obtained from pasture milks were creamier, less firm, less elastic, with a distinctive yellow-red color. These differences were related to the compositional differences found between the milks from pasture and indoor feeding systems. They were attributed to the higher oleic:palmitic acid ratio and PUFA content, along with altered cheese and milk mineral profile (Ca and P) derived from pasture. Evaluating the effect of pasture vs TMR diets on the sensory properties of Caciocavallo Silano cheese; Esposito, et al. [101] reported that pasture derived cheese was yellower, had lower intensity for bitter flavor, smoked and butter odor and higher intensity for spicy flavor. Carpino, et al. [192] found a number of odor-active VOCs in Ragusano cheeses unique to pasture diets (of plant origin) that were absent in the same cheese variety made from milk of TMR diets. These compounds were created through the oxidation processes that may have occurred in the grass during foraging and ingestion by the cow during grazing and transferred to the cheese.

## 10. Conclusions

Several factors dictate the feeding system used on dairy farms for milk production in different regions of the world, including weather, land availability, and the dairy cows’ energy requirements. In Ireland, a pasture-based feeding system prevails due to fertile soils, a temperate climate and abundant rainfall that favor the growth of grass. This pasture feeding supports the competitive stance of Ireland as well as other countries/dairy farmers adopting a pasture-based feeding system among dairy producers. The overall composition and quality of the diet offered through pasture or TMR feeding systems varies considerably and these differences have a significant effect on the composition and quality of milk. Milk derived from cows fed pasture-based diets, is reported to have a higher fat and protein content with improved nutritional status (higher PUFA concentrations and better n-6:n-3 ratio) compared to milk that is derived from a TMR feeding system. Although TMR diets might provide improved ruminal conditions to enhance milk fat and protein yields, TMR can also produce milks with higher concentrations of SFA. Furthermore, pasture feeding has been demonstrated to increase milk concentrations of a variety of beneficial nutrients including vaccenic acid, CLA, ß-carotene, and α-linolenic acid. Such changes affect the nutritional composition and the sensory characteristics of dairy products produced from milk. Cow feeding system has also been demonstrated to impact the functional characteristics, color and textural properties of milk. The impact of feeding system on the composition of bovine milk and processability has been an active area of research, with that a variety of potential biomarkers of pasture feeding have been identified. Future work to develop robust methods for the verification of pasture derived dairy products will be important as “Grass-Fed” dairy products become more prominent on the shop shelves. 

## Figures and Tables

**Table 1:** Contents of macro and micronutrients in bovine milk.

Component		Concentration	Unit	Reference
**Macronutrients**				
**Lipids**	**Fat**	**3.42–5.27**	**%**	[18,20,35]
	Triglycerides	~98	% of total lipids	[36]
	Long chain FA *(C ≥ 16)*	50–70	% of total FA in fat globule	[34,37]
	Short and medium chain FA *(4 ≤ C ≤ 8; 10 ≤ C ≤ 14)*	30–50		
**Nitrogen**	**Protein**	**2.82–4.49**	%	[18,20,35]
	Caseins	2.32–3.52	%	[18,20]
	*αs_1_-Casein*	1.1		[38,39]
	*αs_2_-Casein*	0.3		
	*β-Casein*	0.9		
	*κ-Casein*	0.3		
	Whey proteins	0.83–3.52		[20,40]
	*β-Lactoglobulin*	0.32		[35,39]
	*α-Lactalbumin*	0.12		
	*Serum albumin*	0.04		
	*Lactoferrin*	0.02–0.3	g/L	[41,42]
	*IgA*	0.04–0.06		
	*IgM*	0.03–0.06		
	*IgG*	0.59		
	*Lysozyme*	0.0014–0.007		
	MFGM proteins	1–2	% of total proteins	[43]
	Non-protein nitrogen (NPN)	0.03–0.2	%	[20,42]
	*Urea*	0.654	mg N/L	[42,44]
	*Creatinine*	0.019		
	*Creatine*	0.355		
	*Uric acid*	0.155	%	
	*α-amino N*	2.20	μg/mL	
**Carbohydrate**	**Lactose**	**0.46–0.53**	g/L	[2,18,20]
**Micronutrients**				
**Macro elements**	Calcium (Ca)	113.58–150.4	mg/100 g	[18,35,39,42]
	Phosphorus (P)	87.04–109.6		
	Potasium (K)	143–152		
	Magnesium (Mg)	9.40–15		
	Sodium (Na)	48–58		
	Chlorine (Cl)	100		
	Sulphur (S)	32		
**Trace elements**	Iodine (I)	0.021		
	Ferrous (Fe)	0.022–0.166		
	Copper (Cu)	0.003–0.06		
	Zinc (Zn)	0.408–0.532		
	Manganese (Mn)	0.002–0.02		
	Selenium (Se)	0.001–0.96		
	Cobalt (Co)	0.002–0.023		
**Fat Soluble Vitamins**	Vitamin A (retinol)	0.4	mg/L	[38,39,42]
	Vitamin D (calciferol)	0.001		
	Vitamin E (tocopherol)	1.0		
	Vitamin K (phylloquinone)	0.004–0.018		
**Water Soluble Vitamins**	Thiamin	40		
	Vitamin B_2_ (riboflavin)	190		
	Niacin	0.08		
	Vitamin B_12_	0.357		
	Vitamin B_6_ (pyridoxine)	0.6		
	Folic acid	0.05–5		
	Pantothenic acid	3.5		
	Biotin	0.03–2		
	Vitamin C	20		
